# Supplementary material for: Serum vitamin D, blood pressure and hypertension risk in the HUNT study using observational and Mendelian randomization approaches
Source: Sci Rep. 2024 Jun 21;14:14312. doi: 10.1038/s41598-024-64649-6 (PMC11192928; doi:10.1038/s41598-024-64649-6)
Supplement: Supplementary file 1 — Supplementary Information. [file 41598_2024_64649_MOESM1_ESM.pdf]

Serum vitamin D, Blood pressure and Hypertension Risk in The HUNT Study using Observational and Mendelian Randomization Approaches

Lin Jiang[1,2]*

Yi-Qian Sun[3,4,5]

Marion Denos[1]

Ben Michael Brumpton[6,7,8]

Yue Chen[9]

Vegard Malmo[2,10]

Eleanor Sanderson[11]

Xiao-Mei Mai[1]

[1]Department of Public Health and Nursing, Faculty of Medicine and Health Science, Norwegian University of Science and Technology, Trondheim, Norway

[2]Clinic of Cardiology, St. Olavs Hospital, Trondheim, Norway

[3]Department of Clinical and Molecular Medicine, Faculty of Medicine and Health Science, Norwegian University of Science and Technology, Trondheim, Norway

[4]Department of Pathology, Clinic of Laboratory Medicine, St. Olavs Hospital, Trondheim University Hospital, Trondheim, Norway

[5]TkMidt-Center for Oral Health Services and Research, Mid-Norway, Trondheim, Norway

[6]Clinic of Medicine, St. Olavs Hospital, Trondheim University Hospital, Trondheim, Norway

[7]K.G. Jebsen Centre for Genetic Epidemiology, Department of Public Health and Nursing, Norwegian University of Science and Technology, Norway

[8]HUNT Research Centre, Department of Public Health and Nursing, Norwegian University of Science and Technology, Levanger, Norway

[9]School of Epidemiology and Public Health, Faculty of Medicine, University of Ottawa, Ottawa, Canada

[10]Department of Circulation and Medical Imaging, Norwegian University of Science and Technology, Trondheim, Norway

[11]MRC Integrative Epidemiology Unit, Population Health Sciences, Bristol Medical School, University of Bristol, Bristol, United Kingdom

***Corresponding author**

Address for correspondence: Department of Public Health and Nursing, Faculty of Medicine and Health Science, Norwegian University of Science and Technology (NTNU), Postbox 8905, MTFS, N-7491 Trondheim, Norway.

E-mail: lin.jiang@ntnu.no

Supplementary text 1

We assessed the third assumption for the MR analyses (test whether there existed horizontal pleiotropy) using SNP-based two-sample methods such as MR-Egger [1], weighted median [2] and MR-Pleiotropy Residual Sum and Outlier (MR-PRESSO) [3] methods in the HUNT population. MR-Egger method estimates the causal effect allowing for the presence of pleiotropy. The intercept term in the MR-Egger regression represents the average pleiotropic effect. A non-zero intercept and the P value of the intercept test <0.05 indicates the presence of horizontal pleiotropy [1]. Weighted median method is another robust method that can provide valid casual estimates even when up to 50 % of the SNPs exhibit pleiotropic effects [2]. MR-PRESSO method is used to identify and correct for potential outliers (P<0.05) [3]. This method is based on a modified regression model that includes a correction term for pleiotropy based on a residual-based approach. Since each method is robust to different patterns of horizontal pleiotropy, a good agreement across all the methods suggests that pleiotropy would not influence the causal estimate.

Supplementary text 2

To provide additional evidence on potential causal associations between serum 25(OH)D levels and SBP, DBP and hypertension, two-sample MR was performed with summary data from multiple genome-wide association studies (GWAS). Specifically, we utilized three sets of serum 25(OH)D-associated single nucleotide polymorphisms (SNPs) as instruments to perform our two-sample MR. The first set of SNPs was 6 serum 25(OH)D associated SNPs retrieved from the updated SUNLIGHT Consortium (n=79,366) by Jiang et al. [4]. We also used 35 SNPs from Zhou et al. [5] and 69 SNPs from Manousaki et al. [6] as instruments for serum 25(OH)D to enhance the robustness of the two-sample MR findings. These two sets of SNPs were retrieved based on genome-wide significance (P-value<$5x10^{-8}$). The set of 35 SNPs was first discovered in recent GWAS for serum 25(OH)D in UK Biobank and replicated with a consistent direction and a P-value <0.05 in the earlier GWAS by the SUNGLIGHT consortium (n=294,770). The set of 69 SNPs was the largest set of SNPs for serum 25(OH)D detected from the largest GWAS for serum 25(OH)D using data from both UK Biobank and updated SUNLIGHT (n=443,734). GWAS by Evengelou et al. for SBP or DBP was the largest GWAS for blood pressure, involving a substantial sample size by 757,601 in UK Biobank [7] and therefore was used for the summary data on the systolic blood pressure (SBP) and diastolic blood pressure (DBP) in our two-sample MR. Additionally, we used the largest GWAS for diagnosed hypertension based on ICD code from FinnGen, which included 42,857 cases and 218,792 controls. This dataset has not been previously used [8]. We chose the set of 6 SNPs as our main instruments in the two-sample MR because these 6 SNPs had clear biological function on serum 25(OH)D and no overlapping of the samples for exposure and outcomes existed. The datasets for the exposure and the outcomes were harmonized for each analysis. Inverse-variance weighted, MR-Egger and weighted median methods were used to present the two-sample MR results. Additionally, we used MR Pleiotropy RESidual Sum and Outlier (MR-PRESSO) method to identify potential outliers in the associations between serum 25(OH)D levels (per 25 nmol/L increase) and SBP, DBP or hypertension. MR-Steiger method was further used to test the validity of the selected SNPs and potential direction between SNP and outcome. No outliers were detected by the 6 SNPs as instruments. The results obtained from the MR-Steiger analysis confirmed the validity of all selected 6 SNPs. When we performed additional two-sample MR with 35 SNPs as instruments, we identified 7 outliers for SBP, 6 outliers for DBP and 4 outliers for hypertension. With 69 SNPs as instruments, we detected 18 outliers for SBP, 15 outliers for DBP and 6 outliers for hypertension. After excluding these outliers SNPs, the results kept similar. The results obtained from the MR-Steiger analysis suggested 3 SNPs for SBP, 4 SNPs for DBP and 7 SNPs for hypertension respectively were invalid instruments when we used the 35 SNPs as instruments. There were 4 invalid SNPs for SBP and DBP as outcomes and 3 for hypertension based on the MR-Steiger analysis when we used the 69 SNPs as instruments. After excluding these invalid SNPs, the results kept similar.

Supplementary text 3

To test whether there existed non-linear causal associations in the MR analyses, both the residual method and the doubly-ranked method were used in the sub-cohort of the HUNT population (n=5854). The residual method is a widely used method for the estimation of localized average causal effects at different levels of the exposure distribution [9]. We used the residuals of serum 25(OH)D to divide the sub-cohort of HUNT population into four equal-sized strata under the assumption that the genetic effect on serum 25(OH)D is constant within each stratum [9,10]. The residual of serum 25(OH)D was calculated as the residual from regression of serum 25(OH)D on the mean-centred genetic risk score [10]. The advantage to use residual of serum 25(OH) D rather than the actual serum 25(OH)D levels to stratify the population is to avoid collider bias [10]. The doubly-ranked method is a non-parametric stratification method [11]. In the doubly-ranked method, the population from the sub-cohort of HUNT Study was divided into four equal-sized strata based on rank of the externally weighted genetic risk score from lowest to highest and then the exposure in each stratum was ranked from lowest to highest levels. This method may give unbiased estimates even when the constant genetic effect assumption is violated [11]. However, in a recent paper by Hamilton et al. [12], it suggested that both methods might have provided biased estimates using age and sex as negative control outcomes within the UK Biobank data. The authors suggested that the biased results with the two non-linear methods might be due to selection bias in the UK Biobank data, or population stratification or unclear problems in the methods. Hence, the observed inverse association between serum 25(OH)D and SBP within the lowest stratum using both methods in our study might also be due to unknown bias.

Supplementary Table 1. Baseline characteristics of participants with and without complete information on genetic data in the HUNT Study

Variables	Population with information on genetic data	Population without information on genetic data
**Number of subjects**	**86,435**	**10,001**
Age (years)	46.0 ± 16.8	51.8 ±20.7
Sex (women), %	53.0	54.3
SBP (mmHg)	133.8 ± 22.0	139.6 ± 27.1
DBP (mmHg)	77.6 ± 12.9	79.7 ±14.4
Number of hypertension cases (%)	36,155 (41.9)	4,152 (47.1)
Body mass index (kg/m²)	26.9 ± 4.4	26.5 ± 4.5
Smoking status, % (never/former/current/unknown)	43.6/22.0/22.3/12.2	47.4/17.6/18.9/16.2
Alcohol consumption (times/month), % (never/1–4/≥5/unknown)	23.5/57.8/12.6/6.1	36.4/45.3/8.7/9.6
Physical activity, % (inactive[1]/active[2]/unknown)	19.0/50.8/30.3	23.0/40.5/36.5

DBP: Diastolic blood pressure; HUNT: The Trøndelag Health Study; SBP: Systolic blood pressure; 25(OH)D: 25-hydroxyvitamin D
Data are given as mean ± standard deviation for continuous variables.

[1]Inactive: no physical activity or only light physical activity ≤2h per week. [2]Active: physical activity level from low to high.

Supplementary Table 2. The associations between externally weighted genetic risk score of serum 25(OH)D and potential confounders in the sub-cohort of the HUNT Study (n=5854)

Variables	Coef[1]	95% CI	P value[2]
Age (years)	0.65	-1.32 to 2.62	0.52
Sex (men vs. women)	0.09	-0.14 to 0.33	0.45
Body mass index (kg/m^2)	0.10	-0.38 to 0.58	0.68
Smoking status (ever vs. never)	-0.18	-0.42 to 0.06	0.15
Alcohol consumption (ever vs. never)	-0.09	-0.47 to 0.29	0.64
Physical activity (active[3] vs. inactive[4])	-0.24	-0.55 to 0.06	0.11
Education (<10 year vs. ≥10 years)	0.05	-0.21 to 0.31	0.71
Social economy difficulty (yes vs. no)	-0.03	-0.33 to 0.27	0.84

CI: Confidence interval; Coef: Coefficient; HUNT: The Trøndelag Health Study; 25(OH)D: 25hydroxyvitamin D

[1]Coefficient was derived from linear regression for continuous variables and from logistic regression for categorical variables corresponding to per 25 nmol/L increase in genetically determined serum 25(OH)D.
[2]Significance of Bonferroni corrected P value was calculated as 0.05/8=0.006.
[3]Active: physical activity level from low to high.
[3]Inactive: no physical activity or only light physical activity ≤2h per week.

Supplementary Table 3. Sensitivity analyses to address potential horizontal pleiotropy for the causal associations of serum 25(OH)D levels (per 25 nmol/L increase) with systolic blood pressure, diastolic blood pressure and risk of hypertension in the HUNT study[1] (n=86,324)

	SBP			DBP			Hypertension		
Methods	Coef[2] (mmHg)	95% CI	P value	Coef[2] (mmHg)	95% CI	P value	OR[2]	95% CI	P value
MR-Egger	-0.56	-2.05 to 0.93	0.46	-0.30	-1.28 to 0.68	0.55	1.09	0.88 to 1.34	0.44
MR –Egger Intercept	Effect size			Effect size			Effect size		
	0.06	-0.08 to 0.20	0.39	0.05	-0.05 to 0.14	0.32	-0.01	-0.02 to 0.01	0.60
Weighted median	0.06	-0.85 to 0.96	0.90	0.15	-0.42 to 0.71	0.61	1.10	0.96 to 1.25	0.17
MR-PRESSO[3]	–	–	–	–	–	–	1.10	0.98 to 1.23	0.14

CI: Confidence interval; Coef: Coefficient; DBP: Diastolic blood pressure; HUNT: The Trøndelag Health Study; MR: Mendelian randomization; MR-PRESSO: MR Pleiotropy RESidual Sum and Outlier method; OR: Odds ratio; SBP: Systolic blood pressure; 25(OH)D: 25-hydroxyvitamin D

[1]SNP-based two sample methods were applied to a sub-cohort consisting of 5854 individuals to investigate the gene-exposure association, while the analysis for gene-outcome associations was performing in the total cohort of 86,324 individuals in the HUNT study.

[2]Coefficient was derived from linear regression for SBP/DBP as outcomes while OR was derived from logistic regression for hypertension as outcome in MR-Egger, weighted median and MR-PRESSO methods. All results corresponded to per 25 nmol/L increase in genetically determined serum 25(OH)D after adjustment for age, sex, batch and 20 PCs.

[3]The results based on the MR-PRESSO method were presented only if outliers were detected. For the associations between serum 25(OH)D levels and systolic blood pressure or diastolic blood pressure, no outliers were detected. One outlier (rs12794714) was detected for the association between serum 25(OH)D levels and hypertension.

Supplementary Table 4. Two-sample MR[1] results for the causal associations of serum 25(OH)D levels with systolic blood pressure, diastolic blood pressure and risk of hypertension using 6 SNPs from Jiang et al. as instruments for serum 25(OH)D levels

	**SBP (n=757,601)**			**DBP (n=757,601)**			**Hypertension (cases=42,857 controls=218,792)**		
Methods	Coef[2] (mmHg)	95% CI	P value	Coef[2] (mmHg)	95% CI	P value	OR[2]	95% CI	P value
Inverse-variance weighted	-0.60	-1.95 to 0.74	0.38	-0.22	-1.19 to 0.74	0.65	1.02	0.88 to 1.19	0.74
MR-Egger	-0.19	-2.80 to 2.43	0.89	0.55	-1.28 to 2.39	0.60	1.05	0.77 to 1.42	0.78
MR –Egger Intercept	Effect size			Effect size			Effect size		
	-0.02	-0.14 to 0.09	0.73	-0.05	-0.14 to 0.05	0.40	-0.00	-0.01 to 0.01	0.88
Weighted median	-0.53	-1.25 to 0.20	0.14	0.02	-0.38 to 0.41	0.93	1.03	0.87 to 1.21	0.70

CI: Confidence interval; Coef: Coefficient; DBP: Diastolic blood pressure; MR: Mendelian randomization; OR: Odds ratio; SBP: Systolic blood pressure; SNP: Single Nucleotide Polymorphism; 25(OH)D: 25-hydroxyvitamin D

[1]Two-sample MR was performed with summary data from different genome-wide association studies (GWAS) sources: the Underlying Genetic Determinants of Vitamin D and Highly Related Traits (SUNLIGHT) Consortium for serum 25(OH)D (n=79,366) by Jiang et al. [4], the UK Biobank GWAS for SBP and DBP (n=757,601) by Evengelou et al.[7], and the GWAS for hypertension from FinnGen, which included 42,857 cases and 218,792 controls [8]. The datasets for the exposure and the outcomes were harmonized for each analysis.

[2]Coefficient was derived from linear regression for SBP and DBP as outcomes while OR was derived from logistic regression for hypertension as outcome corresponding to 1 standard deviation increase in genetically determined log-transformed serum 25(OH)D in inverse-variance weighted, MR-Egger and weighted median methods.

Supplementary Table 5. Two-sample MR[1] results for the causal associations of serum 25(OH)D levels with systolic blood pressure, diastolic blood pressure and risk of hypertension using two sets of instruments selected from recent large GWASs for serum 25(OH)D levels

**Instruments**		**SBP (n=757,601)**			**DBP (n=757,601)**			**Hypertension (cases=42,857 controls=218,792)**		
35 SNPs[1]	Methods	Coef[2] (mmHg)	95% CI	P value	Coef[2] (mmHg)	95% CI	P value	OR[2]	95% CI	P value
	Inverse-variance weighted	-0.19	-0.17 to 1.10	0.67	-0.08	-0.54 to 0.71	0.79	1.00	0.89 to 1.13	0.95
	MR-Egger	-0.29	-0.87 to1.45	0.63	-0.12	-0.92 to 0.67	0.75	1.02	0.87 to 1.19	0.81
	MR –Egger Intercept	Effect size			Effect size			Effect size		
		0.01	-0.06 to 0.08	0.79	0.00	-0.04 to 0.05	0.85	-0.00	-0.01 to 0.01	0.74
	Weighted median	-0.15	-0.44 to 0.44	0.28	-0.02	-0.14 to 0.19	0.77	1.02	0.96 to 1.09	0.54
69 SNPs[1]	Inverse-variance weighted	-0.31	-1,28 to 0.66	0.53	-0.03	-0.63 to 0.58	0.93	0.97	0.86 to 1.10	0.65
	MR-Egger	-0.57	-1.86 to 0.73	0.40	-0.27	-1.07 to 0.53	0.51	1.01	0.86 to 1.19	0.90
	MR –Egger Intercept	Effect size			Effect size			Effect size		
		0.01	-0.03 to0.06	0.56	0.01	-0.02 to 0.05	0.36	-0.00	-0.01 to 0.00	0.49
	Weighted median	-0.22	-0.55 to 0.12	0.20	-0.00	-0.19 to 0.18	0.98	1.01	0.94 to 1.08	0.87

CI: Confidence interval; Coef: Coefficient; DBP: Diastolic blood pressure; GWAS: Genome-wide association study; MR: Mendelian randomization; OR: Odds ratio; SBP: Systolic blood pressure; SNP: Single Nucleotide Polymorphism; 25(OH)D: 25-hydroxyvitamin D

[1]Additional two-sample MR studies were performed using another two sets of instruments selected from recent large GWASs for serum 25(OH)D levels: 35 single nucleotide polymorphism (SNP)s from the study by Zhou et al. (n=294,970) [5] and 69 SNPs from the study by Manousaki et al. (n=443,734) [6]. In these two-sample MRs, we employed the same summary data for gene to outcome associations as in a prior two-sample MR, which used 6 SNPs from Jiang et al. as instruments for serum 25(OH)D levels: the UK Biobank GWAS for SBP and DBP (n=757,601) by Evengelou et al.[7], and the GWAS for hypertension from FinnGen, which included 42,857 cases and 218,792 controls [8]. The datasets for the exposure and the outcomes were harmonized for each analysis.

[2]Coefficient was derived from linear regression for SBP and DBP as outcomes while OR was derived from logistic regression for hypertension as outcome corresponding to 1 standard deviation increase in genetically determined log-transformed serum 25(OH)D in inverse-variance weighted, MR-Egger and weighted median methods.

.

Supplementary Table 6. Non-linear causal associations between serum 25(OH)D levels (per 25 nmol/L increase) with systolic blood pressure, diastolic blood pressure and risk of hypertension using different non-linear MR methods in the sub-cohort of HUNT study (n=5854)

**Outcomes**		**Residual method**		**Doubly-ranked method**	
	Stratum	Coef (mmHg)/OR[1]	95% CI	Coef (mmHg)/OR[1]	95% CI
SBP	1 (lowest)	-6.75	-13.23 to -0.27	-7.05	-14.03 to -0.08
	2	-5.18	-11.40 to 1.03	-7.60	-14.82 to -0.38
	3	-1.46	-7.52 to 4.61	-0.32	-7.35 to 6.71
	4 (highest)	1.34	-5.10 to 7.78	1.83	-4.93 to 8.59
DBP	1 (lowest)	-1.41	-5.47 to 2.65	-3.96	-8.33 to 0.42
	2	-3.98	-7.62 to -0.33	-3.22	-7.45 to 1.02
	3	-0.88	-4.54 to 2.79	-1.52	-5.66 to 2.63
	4 (highest)	-1.51	-5.30 to 2.28	-0.21	-4.27 to 3.85
Hypertension	1 (lowest)	0.89	0.41 to 1.96	1.14	0.49 to 2.62
	2	0.47	0.22 to 1.00	0.55	0.23 to 1.32
	3	1.26	0.58 to 2.73	0.80	0.34 to 1.87
	4(highest)	0.93	0.42 to 2.09	0.93	0.39 to 2.20

CI: Confidence interval; Coef: Coefficient; DBP: Diastolic blood pressure; HUNT: The Trøndelag Health Study; MR: Mendelian randomization; OR: Odds ratio; SBP: Systolic blood pressure; 25(OH)D: 25-hydroxyvitamin D

[1]Coefficient was derived from linear regression for SBP and DBP as outcomes and OR from logistic regression for hypertension as outcome corresponding to per 25 nmol/L increase in genetically determined serum 25(OH)D with adjustment for age, sex, batch and 20 PCs within each stra

Supplementary Table 7. Characteristics of 19 SNPs included in the externally weighted genetic risk score for serum 25(OH)D in the HUNT Study (n=86,324)

SNP	Chr	Position_b37	Gene	Effective allele	Alternative allele	External weights[1]
rs1352846	4	72617775	GC	G	A	0.172
rs7041	4	72618334	GC	C	A	-0.045
rs4694431	4	72634343	GC	T	C	-0.034
rs16913816	11	14339328	CYP2R1	A	G	-0.031
rs117913124	11	14900931	CYP2R1	A	G	0.503
rs117576073	11	14912573	CYP2R1	T	G	0.246
rs12794714	11	14913575	CYP2R1	A	G	0.139
rs202122669	11	14913645	CYP2R1	A	G	-0.615
rs187639972	11	14913900	CYP2R1	C	G	-0.36
rs117115472	11	14941652	CYP2R1	G	C	0.148
rs139168803	11	71157867	DHCR7	A	G	-0.188
rs12573951	11	71158672	DHCR7	G	A	-0.045
rs7928249	11	71161063	DHCR7	G	A	-0.131
rs549000212	11	71180762	DHCR7	A	C	-0.364
rs4081429	11	71290740	DHCR7	C	A	0.017
rs6123359	20	52714706	CYP24A1	G	A	-0.026
rs6127099	20	52731402	CYP24A1	T	A	0.013
rs2585442	20	52737123	CYP24A1	G	C	-0.025
rs2762942	20	52788925	CYP24A1	A	G	-0.053

Chr: Chromosome number; HUNT: The Trøndelag Health Study; Position_b37: Base pair position from Genome Reference Consortium Human Build 37 (GRCh37); SNP: Single Nucleotide Polymorphism

[1]The external weights were reported beta coefficient (β) for association with serum 25(OH)D levels from the study by Emerging Risk Factors Collaboration ECVDVDSC[10] .

Supplementary Table 8. Characteristics of SNPs used as genetic instruments for serum 25(OH)D levels from different GWASs in two-sample MR

GWASs	SNP	Chr	Position_b37	Gene	Effective allele	Alternative allele	Beta	SE	P	N
Jiang et al.[1]	rs3755967	4	72609398	GC	T	C	-0,089	0,0023	4.74E-343	79366
(n=6)	rs12785878	11	71167449	NADSYN1/DHCR7	T	G	0,036	0,0022	3,8E-62	79366
	rs10741657	11	14914878	CYP2R1	A	G	0,031	0,0022	2,05E-46	79366
	rs17216707	20	52732362	CYP24A1	T	C	0,026	0,0027	8,14E-23	79366
	rs10745742	12	96358529	AMDHD1	T	C	0,017	0,0022	1,88E-14	79366
	rs8018720	14	39556185	SEC23A	C	G	-0,017	0,0029	4,72E-09	79366
Zhou et al.[2]	rs6671730	1	2339139	PEX10	G	A	0,0061	0.0023	0.006652	29497
(n=35)	rs35408430	1	17560195	PADI1	C	T	0,023699	0.00564768	0.00002715	29497
	rs7522116	1	41835685	FOXO6	C	T	0,011673	0.00540416	0.03077654	29497
	rs7528419	1	109817192	CELSR2	G	A	0,017905	0.00644566	0.0054732	29497
	rs1933064	1	152301576	FLG-AS1	A	G	0,015507	0.00539368	0.00404027	29497
	rs76798800	1	154994978	DCST2	G	T	0,01739	0.00617041	0.00482841	29497
	rs6672758	1	230303512	GALNT2	T	C	0,01563	0.00666121	0.01895423	29497
	rs727857	2	58981967	LINC01122	G	A	0,010913	0.00550772	0.04754487	29497
	rs1047891	2	211540507	CPS1	C	A	0,012657	0.00572581	0.02706743	29497
	rs2012736	2	234622379	UGT1A5, UGT1A6, UGT1A7, UGT1A8, UGT1A9, UGT1A10	C	A	0,038441	0.01038238	0.00021344	29497
	rs6782190	3	85639672	CADM2	G	A	0,020672	0.00562451	0.00023756	29497
	rs705117	4	72608115	GC	C	T	0,026943	0.00744137	0.00029382	29497
	rs1352846	4	72617775	GC	A	G	0,222184	0.00589771	1.40E-310	29497
	rs78151190	6	25619007	CARMIL1 (LRRC16A)	A	C	0,018712	0.00829773	0.02413132	29497
	rs75741381	7	100809458	VGF	C	G	0,021447	0.00736197	0.00357669	29497
	rs12056768	8	116988527	LINC00536	T	G	0,017662	0.00545433	0.00120331	29497
	rs77532868	10	88081438	GRID1	T	C	0,028055	0.01353113	0.03813628	29497

	rs12794714	11	14913575	CYP2R1	G	A	0,070249	0.00540376	1.22E-38	29497
	rs61891388	11	66079818	RP11-867G23.13	G	T	0,011425	0.00538961	0.03401532	29497
	rs1660839	11	71094232	AP002387.1	A	G	0,014173	0.00623639	0.02304867	29497
	rs12803256	11	71132868	AP002387.1	G	A	0,083912	0.00602549	4.39E-44	29497
	rs12798050	11	71223256	S100A11P3	T	C	0,0348	0.0024	1.00E-47	29497
	rs72997623	11	75488054	DGAT2	A	C	0,020016	0.00937765	0.03280964	29497
	rs1149605	11	76485216	RP11-21L23.4	C	T	0,020979	0.0072426	0.00377288	29497
	rs10859995	12	96375682	HAL	T	C	0,036551	0.00540543	1.36E-11	29497
	rs8018720	14	39556185	SEC23A	G	C	0,040852	0.00705183	6.91E-09	29497
	rs261291	15	58680178	ALDH1A2	T	C	0,011347	0.0056366	0.04410853	29497
	rs77924615	16	20392332	PDILT	G	A	0,019554	0.00670691	0.00355194	29497
	rs212100	19	48376995	SULT2A1	T	C	0,019388	0.00719712	0.00706453	29497
	rs10426	19	51517798	KLK10	A	G	0,014645	0.0065379	0.02509092	29497
	rs6123359	20	52714706	BCAS1	G	A	0,037364	0.00940288	0.00007078	29497
	rs17216707	20	52732362	CYP24A1	T	C	0,06469	0.0066412	2.02E-22	29497
	rs2585442	20	52737123	CYP24A1	G	C	0,038148	0.00635676	1.96E-09	29497
	rs2762943	20	52790786	CYP24A1	G	T	0,032534	0.01442103	0.02406994	29497
	rs2074735	22	31535872	PLA2G3	C	G	0,021368	0.01054517	0.04273241	29497
Manousaki et al.[3]	rs6698680	1	2329661	RER1	A	G	0,011928	0,001947	8,99189E-10	443734
(n=69)	rs3750296	1	17559656	PADI1	G	C	0,020818	0,002042	2,08945E-24	443734
	rs7519574	1	34726552	RP4-657M3.2	A	G	0,016991	0,002536	2,08532E-11	443734
	rs56044892	1	41830086	FOXO6	T	C	0,015388	0,00244	2,8531E-10	443734
	rs2934744	1	63048045	DOCK7	C	A	0,022405	0,002119	3,95946E-26	443734
	rs7528419	1	109817192	CELSR2	G	A	0,019031	0,002321	2,41423E-16	443734
	rs3768013	1	150815411	ARNT	G	A	0,01488	0,002011	1,369E-13	443734
	rs144613541	1	152270875	FLG	G	A	0,015413	0,002244	6,4863E-12	443734
	rs11264360	1	155284586	FDPS	A	T	0,018008	0,002286	3,33955E-15	443734
	rs867772	1	220972343	MARC_1	A	G	0,013838	0,002091	3,64371E-11	443734
	rs10127775	1	230295789	GALNT2	T	A	0,011801	0,001997	3,43431E-09	443734

rs12997242	2	21381177	TDRD15	G	A	0,012512	0,001972	2,22675E-10	443734
rs11127048	2	27752463	GCKR	A	G	0,018107	0,002038	6,40987E-19	443734
rs6724965	2	101440151	NPAS2	A	G	0,016541	0,002573	1,28715E-10	443734
rs7569755	2	118648261	HTR5BP	A	G	0,013923	0,002142	8,032E-11	443734
rs1047891	2	211540507	CPS1	C	A	0,014168	0,002088	1,15733E-11	443734
rs2011425	2	234627608	UGT1A4	T	G	0,046356	0,00361	9,66218E-38	443734
rs7650253	3	49431160	RHOA	A	T	0,014562	0,002282	1,75655E-10	443734
rs1972994	3	85631142	CADM2	A	T	0,017509	0,002036	7,99211E-18	443734
rs6438900	3	125148287	MRPL3	G	C	0,013584	0,002221	9,58547E-10	443734
rs6773343	3	141825598	TFDP2	T	C	0,01268	0,002171	5,20049E-09	443734
rs78649910	4	3482213	DOK7	T	A	0,018331	0,003122	4,31722E-09	443734
rs7699711	4	69947596	UGT2B7	G	T	0,02864	0,001949	6,96908E-49	443734
rs11723621	4	72615362	GC	A	G	0,18693	0,002121	3.0E-1443	443734
rs58073039	4	88287363	HSD17B11	A	G	0,014119	0,002109	2,162E-11	443734
rs7718395	5	118652574	TNFAIP8	G	C	0,012632	0,002096	1,67323E-09	443734
rs3822868	6	131934986	MED23	G	A	0,021918	0,002745	1,40865E-15	443734
rs111529171	7	21571932	DNAH11	G	C	0,015488	0,002369	6,24388E-11	443734
rs1011468	7	104613791	LINC01004	G	A	0,013795	0,001946	1,35182E-12	443734
rs1858889	7	107117447	COG5	C	A	0,012836	0,001942	3,8515E-11	443734
rs804280	8	11612698	GATA4	A	C	0,013033	0,001978	4,42862E-11	443734
rs34726834	8	25889606	EBF2	T	C	0,013824	0,002239	6,65057E-10	443734
rs7828742	8	116960729	LINC00536	A	G	0,02193	0,00199	3,05718E-28	443734
rs10818769	9	125719923	RABGAP1	C	G	0,016972	0,00287	3,34733E-09	443734
rs532436	9	136149830	ABO	G	A	0,015051	0,002515	2,17065E-09	443734
rs10887718	10	82042624	MAT1A	C	T	0,012476	0,001946	1,44474E-10	443734
rs10832289	11	14669496	PDE3B	A	T	0,068522	0,001965	2,0288E-266	443734
rs523583	11	66070146	TMEM151A	C	A	0,012174	0,001963	5,58451E-10	443734
rs12803256	11	71132868	FLJ42102	G	A	0,100325	0,002325	1.3E-378	443734
rs1149605	11	76485216	RP11-21L23.4	C	T	0,01928	0,002577	7,34412E-14	443734
rs964184	11	116648917	ZPR1	C	G	0,03977	0,002858	5,11267E-44	443734

rs2847500	11	120114421	POU2F3	G	A	0,021129	0,002949	7,78993E-13	443734
rs12317268	12	21352541	SLCO1B1	A	G	0,018528	0,002717	9,14941E-12	443734
rs9668081	12	38602911	FAM166AP9	T	C	0,0116	0,001988	5,37873E-09	443734
rs10859995	12	96375682	HAL	T	C	0,039396	0,001971	7,02959E-89	443734
rs8018720	14	39556185	SEC23A	G	C	0,031949	0,002546	4,0407E-36	443734
rs261291	15	58680178	ALDH1A2	T	C	0,022414	0,002033	2,89242E-28	443734
rs1800588	15	58723675	LIPC	C	T	0,029769	0,002366	2,65246E-36	443734
rs17765311	15	63789952	AC007950.2	A	C	0,01515	0,002047	1,35087E-13	443734
rs62007299	15	77711719	PEAK1	G	A	0,014437	0,002145	1,69039E-11	443734
rs8063706	16	11909552	BCAR4	T	A	0,012968	0,002198	3,63702E-09	443734
rs77924615	16	20392332	PDILT	G	A	0,015792	0,002464	1,4639E-10	443734
rs71383766	16	30930233	HMG20A	A	G	0,012569	0,002065	1,15274E-09	443734
rs1800775	16	56995236	CETP	C	A	0,016619	0,00195	1,56059E-17	443734
rs2909218	17	66464546	RP11-120M18.2	T	C	0,016894	0,002418	2,81295E-12	443734
rs8091117	18	28919794	DSG1	C	A	0,024071	0,003943	1,02966E-09	443734
rs2037511	18	61366207	SERPINB11	A	G	0,016025	0,002618	9,29404E-10	443734
rs57631352	19	4338173	STAP2	A	G	0,012866	0,002128	1,48438E-09	443734
rs73015021	19	11192915	LDLR	G	A	0,023034	0,002983	1,14737E-14	443734
rs58542926	19	19379549	TM6SF2	T	C	0,032488	0,00367	8,57215E-19	443734
rs3814995	19	36342212	NPHS1	C	T	0,014733	0,002109	2,83287E-12	443734
rs157595	19	45425460	APOC1	A	G	0,015581	0,00205	2,95017E-14	443734
rs112285002	19	48374320	SULT2A1	T	C	0,060321	0,002701	1,773E-110	443734
rs10426	19	51517798	KLK10	A	G	0,025226	0,002382	3,30671E-26	443734
rs8103262	19	53065814	ZNF808	C	T	0,012519	0,002114	3,18148E-09	443734
rs6127099	20	52731402	RP13-379L11.3	A	T	0,036797	0,002219	9,29648E-62	443734
rs2229742	21	16339172	NRIP1	G	C	0,025738	0,00319	7,12612E-16	443734
rs2074735	22	31535872	PLA2G3	C	G	0,027256	0,003969	6,54646E-12	443734
rs960596	22	41393520	SCUBE1	T	C	0,012414	0,002076	2,23455E-09	443734

Chr: Chromosome number; MR: Mendelian randomization; GWAS: Genome-wide association study; Position_b37: Base pair position from Genome Reference Consortium Human Build 37 (GRCh37); SE: Standard error; SNP: Single Nucleotide Polymorphism; 25(OH)D: 25-hydrovitamin D

[1]Six genome-wide significant vitamin D SNPs were selected from the Underlying Genetic Determinants of Vitamin D and Highly Related Traits (SUNLIGHT) Consortium for serum 25(OH)D (n=79,366) by Jiang et al.[4]. These 6 SNPs were used as the primary instruments in the two-sample MR due to the clear biological relevance to serum 25(OH)D (mean F statistics was 358) and no overlap between the exposure and outcome GWAS datasets.
[2]We employed 35 genome-wide significant vitamin D SNPs obtained from Zhou et al. to conduct supplementary two-sample MR analyses [5]. The set of 35 SNPs was first discovered in a recent GWAS for serum 25(OH)D in UK Biobank and replicated with a consistent direction and a P-value <0.05 in the earlier GWAS by the Underlying Genetic Determinants of Vitamin D and Highly Related Traits (SUNLIGHT) consortium (n=294,770) [5]. The mean F statistics were 81.
[3]The 69 SNPs from Manousaki et al. were common SNPs selected based on genome-wide significance in the largest GWAS on serum 25(OH)D using data from both UK Biobank and updated SUNLIGHT (n=443,734) [5,6]. The mean F statistics were 279.

References

1. Bowden J, Davey Smith G, Burgess S. Mendelian randomization with invalid instruments: effect estimation and bias detection through Egger regression. **Int J Epidemiol**. 2015;44(2):512-525.
2. Bowden J, Davey Smith G, Haycock PC, Burgess S. Consistent Estimation in Mendelian Randomization with Some Invalid Instruments Using a Weighted Median Estimator. **Genet Epidemiol**. 2016;40(4):304-314. doi:https://doi.org/10.1002/gepi.21965
3. Verbanck M, Chen CY, Neale B, Do R. Detection of widespread horizontal pleiotropy in causal relationships inferred from Mendelian randomization between complex traits and diseases. **Nat Genet**. May 2018;50(5):693-698. doi:10.1038/s41588-018-0099-7
4. Jiang X, O'Reilly PF, Aschard H, et al. Genome-wide association study in 79,366 European-ancestry individuals informs the genetic architecture of 25-hydroxyvitamin D levels. **Nat Commun**. Jan 17 2018;9(1):260. doi:10.1038/s41467-017-02662-2
5. Zhou A, Selvanayagam JB, Hyppönen E. Non-linear Mendelian randomization analyses support a role for vitamin D deficiency in cardiovascular disease risk. **Eur Heart J**. 2022;7;43(18):1731-1739doi:10.1093/eurheartj/ehab809
6. Manousaki D, Mitchell R, Dudding T, et al. Genome-wide Association Study for Vitamin D Levels Reveals 69 Independent Loci. **Am J Hum Genet**. Mar 5 2020;106(3):327-337. doi:10.1016/j.ajhg.2020.01.017
7. Evangelou E, Warren HR, Mosen-Ansorena D, et al. Genetic analysis of over 1 million people identifies 535 new loci associated with blood pressure traits. **Nat Genet**. 2018/10/01 2018;50(10):1412-1425. doi:10.1038/s41588-018-0205-x
8. Kurki MI, Karjalainen J, Palta P, et al. FinnGen provides genetic insights from a well-phenotyped isolated population. **Nature**. 2023/01/01 2023;613(7944):508-518. doi:10.1038/s41586-022-05473-8
9. Burgess S, Davies NM, Thompson SG. Instrumental variable analysis with a nonlinear exposure–outcome relationship. **Epidemiology (Cambridge, Mass)**. 2014;25(6):877.
10. Estimating dose-response relationships for vitamin D with coronary heart disease, stroke, and all-cause mortality: observational and Mendelian randomisation analyses. **Lancet Diabetes Endocrinol**. Jan 2024;12(1):e2-e11. doi:10.1016/s2213-8587(23)00287-5
11. Burgess S. Violation of the constant genetic effect assumption can result in biased estimates for non-linear Mendelian randomization. **Humam Heredity**. 2023;88(1):79-90. doi:10.1159/000531659
12. Hamilton FW, Hughes DA, Spiller W, Tilling K, Smith GD. Non-linear mendelian randomization: evaluation of biases using negative controls with a focus on BMI and Vitamin D. **medRxiv**. 2023:08.21.23293658. doi:10.1101/2023.08.21.23293658
